# Micro lesion effect of pallidal deep‑brain stimulation for meige syndrome

**DOI:** 10.1038/s41598-022-23156-2

**Published:** 2022-11-21

**Authors:** Jiayu Liu, Hu Ding, Ke Xu, Dongliang Wang, Jia Ouyang, Zhi Liu, Ruen Liu

**Affiliations:** Department of Neurosurgery, Peking University People’s Hospital, 11Th Xizhimen South St., Beijing, 100044 China

**Keywords:** Neurology, Neurological disorders

## Abstract

To analyse the microlesion effect (MLE) in the globus pallidus interna (GPi) of deep brain stimulation (DBS) in patients with Meige syndrome. Thirty-two patients with primary Meige syndrome who underwent GPi-DBS in this study. Burke–Fahn–Marsden Dystonia Rating Scale scores (BFMDRS-M) were obtained for the evaluation of clinical symptoms at 3 days before DBS (baseline), 24 h after DBS surgery, once weekly for 1 month until electrical stimulation, 6 months postoperatively and 12 months after surgery. Twenty-seven patients had MLE after GPi-DBS. The mean time of BFMDRS-M scores maximal improvement from MLE was 35.9 h postoperatively (range, 24–48 h), and the mean scores improved by 49.35 ± 18.16%. At 12 months after surgery, the mean BFMDRS-M scores improved by 50.28 ± 29.70%. There was a positive correlation between the magnitude of MLE and the motor score at 12 months after GPi-DBS (R2 = 0.335, *p* < 0.05). However, there was no correlation between the duration of MLE and DBS improvement. Most Meige syndrome patients who underwent GPi-DBS and had MLE benefited from MLE. For Meige syndrome, MLE might be a predictive factor for patient clinical symptom improvement from DBS.

## Introduction

Meige syndrome is a kind of dystonia that tends to occur in middle-aged and elderly women. It was named after Henry Meige, a French neurologist, who first summarized this craniocervical segmental dystonia with bilateral blepharospasm and with oromandibular dystonia as the core symptom^1,2^. There are few cases of spontaneous remission of Meige syndrome.Slow progression is also characteristic of Meige syndrome. In severe cases, it may even lead to functional blindness, temporomandibular joint dislocation and respiratory muscle spasm, which further has a considerable impact on the quality of life of patients^1^.

Deep brain stimulation (DBS) is performed by surgically implanting stimulation electrodes to specific targets in the deep brain, which can continuously generate perielectrode electrical activity via electrical and neurochemical mechanisms, and modulate abnormal neural network activity and plasticity, and regulate the motor circuit to treat dystonia^2,3^. In recent years, a large number of studies have proposed that DBS is not only improve the movement disorders of patients but also have a positive impact on their mood, sleep, life function and quality^4–7^.The microlesion effect (MLE) has been reported in Parkinson's disease in previous studies^8,9^. The mechanism of MLE may be caused by microscopic haematoma and oedema caused by electrode implantation, which induces the proliferation of local microglia. This effect occurs early in the postoperative period and is often manifested as an improvement in the patient's symptoms^10^. Studies have shown that MLE might be a predictor of the effectiveness of subthalamic deep brain stimulation for Parkinson's disease^11,12^.

To our knowledge, there are few studies on MLE in patients with Meige syndrome.. In addition, whether MLE in the globus pallidus internus (GPi) is a predictor for Meige syndrome patients remains unknown. The aim of this study was to analyse the MLE in GPi-DBS in patients with Meige syndrome and to determine whether the magnitude of MLE could help predict the clinical symptom improvement of DBS in these patients.

## Results

### Patients

A total of 32 patients with Meige syndrome were examined, including 27 patients who had MLE and 5 patients who did not have MLE. The clinical characteristics of the two groups of patients were similar at baseline in terms of age (59.22 ± 8.76 years in the MLE group vs. 61.00 ± 10.37 years in the non-MLE group), female sex (77.78% vs. 80.00%) and disease duration (5.15 ± 4.53 years vs. 3.60 ± 2.51 years). Baseline functional status (BFMDRS-M scores) characteristics did not significantly differ between the two groups (Table 1).

**Table 1:** Baseline characteristics of the patients.

Characteristic*	MLE (n = 27)	Non-MLE (n = 5)	*P* value†
Age -yr	59.22 ± 8.76	61.00 ± 10.37	0.69
Disease duration – yr	5.15 ± 4.53	3.60 ± 2.51	0.75
Baseline BFMDRS-M (range,0–40) §	10.65 ± 4.63	8.10 ± 3.96	0.29
Female sex—no. (%)	21 (77.78)	4 (80.00)	1.00

*Plus–minus values are means ± SD·

^†^P values were calculated with the use of Student’s t-test or analysis of variance for continuous variables and Fisher’s exact test for categorical variables.

^§^A higher score indicates worse functioning.

### Micro Lesion Effect

**Figure 1:**
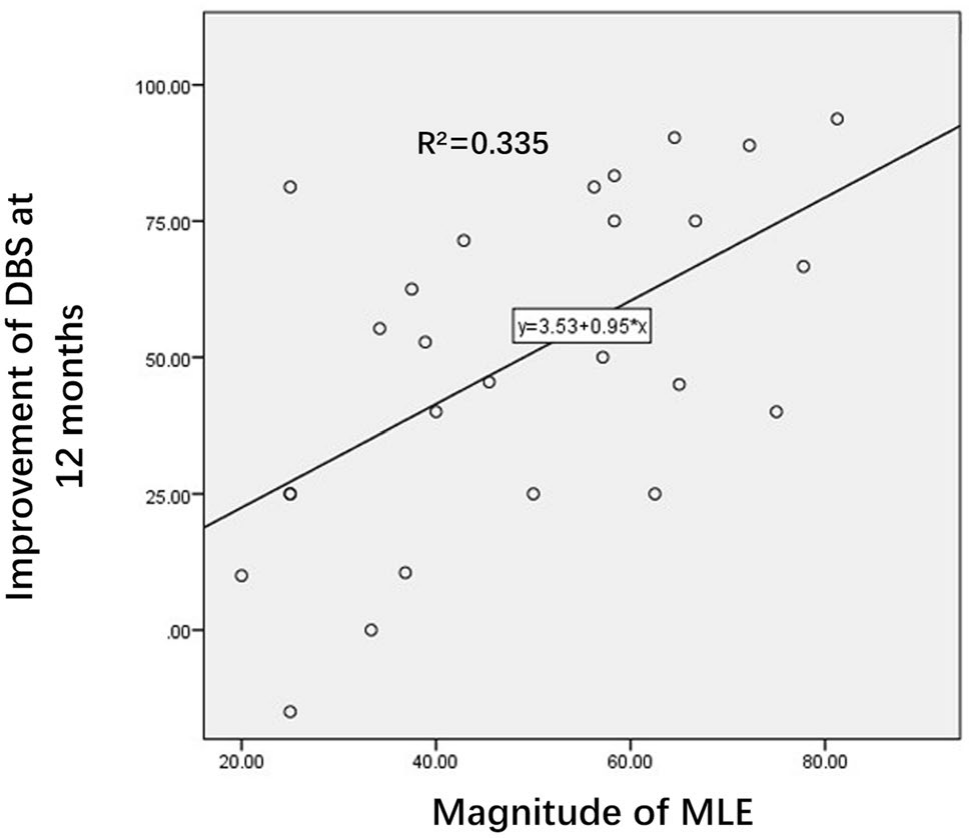
Correlation between the motor score and magnitude of MLE. There was a positive correlation between the improvement of the BFMDRS-M at 12 months following DBS and the magnitude of MLE.

In the MLE group, the MLE presented within the first 48 h after GPi-DBS, and the mean BFMDRS-M scores improved by 49.35 ± 18.16%. The mean time of the BFMDRS-M score maximal improvement from MLE was 35.9 h postoperatively (range, 24–48 h). At 6 months after GPi-DBS, the mean BFMDRS-M scores improved by 46.42 ± 33.68%. At 12 months after GPi-DBS, the mean BFMDRS-M scores improved by 50.28 ± 29.70%. In the MLE group, compared with the baseline, the BFMDRS-M scores were improved at both the MLE (t = 10.8; *p* < 0.001) and 6 months (t = 6.69; < 0.001) and 12 months (t = 7.01; *p* < 0.001) postoperatively. (Table 2) A positive correlation between the magnitude of MLE and the motor score after surgery at 12 months was found in this study (R = 0.579, R^2^ = 0.335 and *p* = 0.002, Fig. 1). However, there was no correlation between the magnitude of MLE and the motor score at 6 months after surgery(R = 0.497, R^2^ = 0.247 and *p* = 0.080). There was also no correlation between the duration of MLE and BFMDRS-M scores improvement at MLE (R = 0.082, R^2^ = 0.007 and *p* = 0.683), 6 months (R = 0.042, R^2^ = 0.002 and *p* = 0.836) and 12 months (R = 0.137, R^2^ = 0.019 and *p* = 0.496).

**Table 2:** Results of assessments per and post DBS at GPi in Meige syndrome patients with and without MLE.

Characteristic*	MLE	Non-MLE	*P* value	T value
**Baseline**				
BFMDRS-M§	10.65 ± 4.63	8.10 ± 3.96	0.29	1.15
**MLE**				
BFMDRS-M	5.56 ± 3.55	8.10 ± 3.96	–	–
Mean change at MLE	5.09 ± 2.45	0	–	–
Improvement at MLE	49.35 ± 18.16%	0.00%	–	–
**6 months**				
BFMDRS-M	5.44 ± 4.02	4.70 ± 4.12	–	–
Mean change at 6 months	5.20 ± 4.04	3.40 ± 2.38	0.35	0.96
Improvement at 6 months	46.42 ± 33.68%	43.82 ± 24.69%	0.87	0.16
**12 months**				
BFMDRS-M	5.30 ± 4.60	5.60 ± 2.88	–	–
Mean change at 12 months	5.35 ± 3.97	2.50 ± 1.17	0.005	3.08
Improvement at 12 months	50.28 ± 29.70%	31.42 ± 4.64%	< 0.001	3.10

*Plus–minus values are means ± SD.

^§^A higher score indicates worse functioning.

In the non-MLE group, the MLE was not present after GPi-DBS. At 6 months after GPi-DBS, the mean scores improved by 43.82 ± 24.69%. At 12 months after GPi-DBS, the mean scores improved by 31.42 ± 4.64%. In the non-MLE group, the BFMDRS-M scores were improved at both the MLE and 6 and 12 months postoperatively. In the non-MLE group, the BFMDRS-M scores were also improved at both the 6 months (t = 3.19; *p* = 0.033) and 12 months (t = 4.77; *p* = 0.009) postoperatively. (Table 2) There were significant differences in improvements in the motor scores between the two groups at 12 months (*p* < 0.001) (Table 3).

**Table 3:** Dystonia scores evolution of the patients.

Characteristic*	MLE	Non-MLE
Baseline BFMDRS-M§	10.65 ± 4.63	8.10 ± 3.96
MLE BFMDRS-M§	5.56 ± 3.55	8.10 ± 3.96
T value	10.80	–
*P* value	< 0.001	–
Baseline BFMDRS-M§	10.65 ± 4.63	8.10 ± 3.96
BFMDRS-M at 6 months	5.44 ± 4.02	4.70 ± 4.12
T value	6.69	3.19
P value	< 0.001	0.033
Baseline BFMDRS-M§	10.65 ± 4.63	8.10 ± 3.96
BFMDRS-M at 6 months	5.30 ± 4.60	5.60 ± 2.88
T value	7.01	4.77
*P* value	< 0.001	0.009

*Plus–minus values are means ± SD.

^§^A higher score indicates worse functioning.

## Discussion

The main symptoms of Meige syndrome are bilateral blepharospasm and oromandibular dystonia^14^. Previous studies have confirmed the effectiveness of DBS in treating motor symptoms of Meige syndrome. The mean symptom improvement rate achieved by GPi-DBS for craniocervical dystonia was 31.6% at 6 months postoperatively and increased to 61.8% at 12 months postoperatively^15^. Sako et al.'s GPi-DBS study followed Meige syndrome patients for an average of 49 months, and the improvement rate of motor symptoms reached 84%^16^. Horisawa et al.^17^ achieved a 58.9% symptom improvement rate in the long-term follow-up of 12 patients. Our study also verified the efficacy of GPi-DBS in Meige syndrome movement disorders.

During DBS surgery, microelectrodes are implanted for electrophysiological testing to identify the target and apply stimulation^18^. Electrode implantation may alter the tissue structure around the target and cause symptoms to change before DBS treatment. This phenomenon is called MLE because its performance and mechanism are similar to those of lesion therapy. Studies on the treatment of dystonia with DBS have shown that before DBS stimulation, patients may show varying degrees of symptom improvement due to MLE, but the effect can fade in a period of time. Therefore, DBS startup treatment should be performed after the end of MLE to avoid its impact on the efficacy of DBS^19^. In this study, 27 patients recorded a improvement in BFMDRS-M scores before DBS simulation, which was considered MLE, while the other 5 patients did not record an improvement in BFMDRS-M scores, which was considered non-MLE. MLE usually presents as an improvement in postoperative symptoms, which has been described in patients with Parkinson's disease and dystonia^8–10,20^. Cersosimo et al.^20^ found that MLE appeared within 24 h after surgery and peaked at 38.5 h (24–72 h) on average, with a duration ranging from 7 days to more than 21 days. There have also been reports of symptom improvement lasting more than 6 months in patients without DBS stimulation^8,21^. In our study, the mean time of maximal motor score improvement from MLE was 35.9 h postoperatively.

Since MLE can appear within 1 day after surgery and the postpeak effect gradually decreases, its time course is similar to that of postoperative haematoma or oedema, so the mechanism of MLE may be related to the above changes. This was confirmed by an fMRI study that observed the appearance of local oedema^22^. In addition, pathological studies showed that MLE presented with minimal bleeding, tissue oedema, and axonal sphericity around the stimulation target^23^. However, the duration of MLE varies greatly among individuals, with the longest duration exceeding 6 months, which is difficult to explain by local haematoma or oedema^20^. There are also studies showing that MLE lasting for a long time may be related to the proliferation of nerve cells^24,25^.

**Figure 2:**
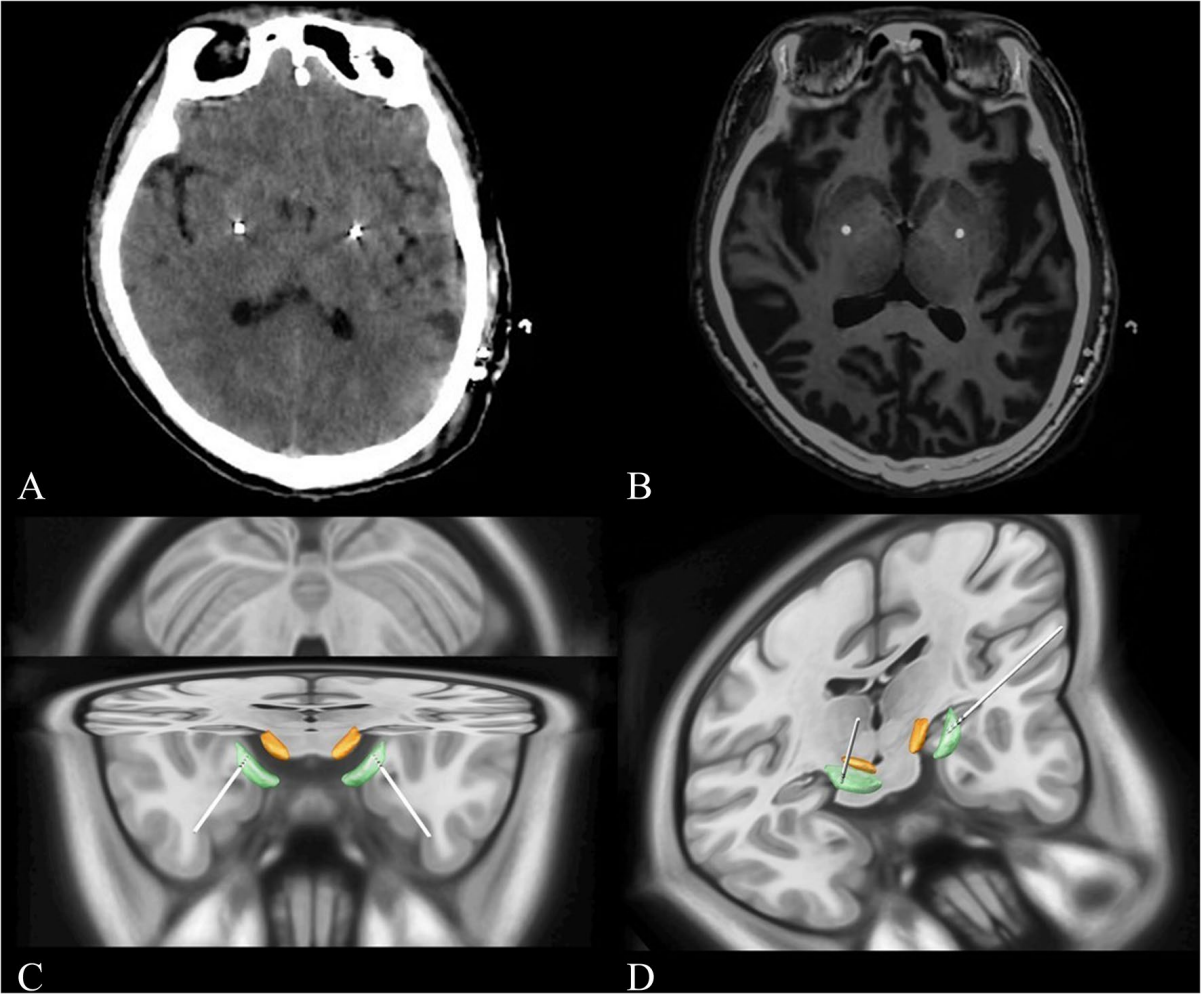
(**A**) Postoperative CT showing the location of electrodes in the internal globus pallidus (GPi); (**B**) Preoperative MR and postoperative CT fusion showing the location of electrodes in the GPi; (**C**) and (**D**)Schematic diagram of 3D reconstruction of the implanted electrodes in the GPi.

At present, there are few studies on the factors influencing the formation of MLE. In this study, intergroup comparison of patients in the MLE group and non-MLE group showed that there were no statistically significant differences in sex, age, preoperative BFM-M baseline score or target selection between the two groups; that is, the above indicators did not affect the formation of MLE. MLE was not observed in 5 patients in the study, and the accuracy of electrode placement should be questioned first. However, it was confirmed by postoperative CT or MRI that the electrode placement was accurate in all patients, and no obvious electrode offset was observed. (Fig. 2) Observable symptom improvement was achieved after the operation, so the cause of electrode placement failure could be excluded. We suggest that these patients may have no symptom improvement or that the amount of symptom improvement (result of MLE) is not reflected by the BFMDRS-M scores.

In our study, we found a positive correlation between the magnitude of MLE and the motor score at 12 months after surgery. For patients with Parkinson's disease, Maltete et al.^11^ proposed in 2008 that such a "microsubthalamotomy effect" was an independent predictor of the long-term efficacy of DBS in Parkinson's disease, and the stronger the MLE under drug withdrawal, the better the long-term efficacy of DBS^12^. However, when taking levodopa and other drugs, there was no significant correlation between MLE and long-term efficacy^26^. This suggests that MLE can be used as a predictor of the long-term efficacy of DBS in Parkinson's disease, but its predictive ability is easily affected by drugs. In addition, although the efficacy of both MLE and DBS in this study showed improvement of patients' symptoms and there was a correlation between the two, the existing data cannot indicate that the treatment principle of MLE and DBS is the same. MLE does not necessarily mean a partial cure or progression of the disease itself but merely that this minor lesion has an effect at the level of symptoms. Pourfar et al.^27^ observed the characteristic glucose metabolism pattern of Parkinson's disease on FDG-PET before surgery, and patients showed improvement in symptoms within several days after electrode implantation, but there was no significant change in PDRP on FDG-PET. However, PDRP changes significantly after DBS is turned on for a period of time.

In summary, this study is the first to find MLE after GPi-DBS treatment in a large number of patients with Meige syndrome. This may be caused by microhaematoma, oedema and local microglial hyperplasia caused by electrode implantation, which will affect the symptoms of patients in the early postoperative period. Therefore, DBS startup should be started approximately 1 month after DBS surgery to reduce the interference of MLE on efficacy evaluation. In addition, this study, for the first time, found a correlation between MLE and DBS long-term efficacy in patients with Meige syndrome. Clinicians can roughly predict the long-term efficacy according to the degree of MLE in patients, which enables doctors and patients to have a more reasonable expectation of efficacy and helps improve the efficiency of program control. It is worth mentioning that STN also is an effective brain target for the treatment of patients with Meige syndrome^5^. In the future, a comparison on how MLE could predict improvement in STN and GPi is necessary. In addition, due to the low incidence of Meige syndrome, the sample size is small. In the future, large sample studies are also necessary.

### Conclusion

In summary, our study found that the magnitude of MLE could help predict the clinical symptom improvement of DBS after 12 months in Meige syndrome patients. Start-up of DBS not to be mixed up with MLE and that clinician should wait until MLE disappear.

## Methods

### Patients

Thirty-two patients with primary Meige syndrome undergoing GPi-DBS were retrospectively analysed between September 2017 and September 2020 at the Department of Neurosurgery, Peking University People’s Hospital. The diagnostic criteria were primarily based on the presence of blepharospasm, oromandibular dystonia and cervical dystonia, increased blink rates and other symptoms^28^.

The inclusion criteria were as follows: (1) primary Meige syndrome was diagnosed by an experienced neurologist; (2) all patients had received systematic and regular treatment, including oral drugs in which the 1st-line therapy included neuroleptics (pimozide, haloperidol) and the 2nd-line therapy included dopamine receptor blocker (tetrabenazine), GABAB receptor agonist (baclofen) and atypical antipsychotics (clozapine)^29^, and local injection of botulinum toxin A for more than 1 year before GPi-DBS, but there was still severe functional impairment; (3) no history of exposure to neuroleptics; (4) normal neurological exam except for dystonia; and (5) normal magnetic resonance imaging of the brain. The exclusion criteria were as follows: (1) patients considered at high risk for elective neurosurgery because of comorbid conditions, such as untreated hypertension or brain MRI showing extensive brain atrophy or small vessel ischaemic disease; and (2) missed follow-up and incomplete clinical data.

Written informed consent was obtained from each participant, and this study was approved by the institutional review board of Peking University People’s Hospital (2020PHB065-01). All methods were carried out in accordance with relevant guidelines and regulations.

### Surgical procedures and DBS programming

The surgical procedure for bilateral GPi-DBS was consistent with previous studies from our team^13,30^. In brief, patients underwent bilateral stereotactic surgery under local anaesthesia. The globus pallidus interna was located by combining stereotactic MRI with microelectrode recording. The GPi is located in the front 2–3 mm from the midpoint of the anteroposterior commissure, 18–22 mm lateral and 6–9 mm below the plane of the anteroposterior commissure. A DBS electrode (model L302, PINS Medical, Beijing, China) and pulse generator (G102R, PINS Medical) were implanted, and the final position of the electrode was confirmed by neuroimaging. (Fig. 2) One month after surgery, stimulation was initiated. The optimal stimulation settings were progressively adjusted according to the patient’s response. The standard pulse setting was 60 μsec in duration at 130 Hz, with the voltage adjusted to the individual patient. In addition, based on each patient’s response to neurostimulation, the parameters could be progressively adjusted at outpatient follow-up or by a telemedical application.

### Evaluation and follow-up

To detect changes in motor function, Burke–Fahn–Marsden Dystonia Rating Scale scores were obtained for the movement subscales (BFMDRS-M)^31^ (scores range from 0–40, with higher scores indicating greater impairment) based on evaluation of video recordings obtained at specific time points: 3 days before DBS (baseline), 24 h after DBS surgery, once weekly for 1 month until electrical stimulation, 6 months postoperatively and 12 months after surgery.

MLE was defined as the improvement in motor scores observed after electrode implantation and before initiating electrical stimulation^20^. The magnitude of MLE was defined as the difference in motor scores between the basal condition (before surgery) and at maximum MLE.

### Statistical analysis

Linear regression analysis was used to test the relationship between the magnitude or duration of the MLE and the postoperative improvement of DBS . Student's t-tests were performed to evaluate data that followed a normal distribution. Significant differences between groups were identified at *P* < 0.05. Numerical variables are expressed as the mean ± SD, and qualitative variables are expressed as the absolute values. With the assistance of SPSS 25.0 (IBM Corp., Armonk, NY, USA, www.ibm.com/cn-zh/spss), statistical analyses were performed.

### Ethics approval

The Ethics Committee of Peking University People’s Hospital (2020PHB065-01).

## Data Availability

The datasets used and/or analysed during the current study available from the corresponding author on reasonable request.
